# Development and external validation of clinical prediction models for pituitary surgery

**DOI:** 10.1016/j.bas.2023.102668

**Published:** 2023-08-28

**Authors:** Olivier Zanier, Matteo Zoli, Victor E. Staartjes, Mohammed O. Alalfi, Federica Guaraldi, Sofia Asioli, Arianna Rustici, Ernesto Pasquini, Marco Faustini-Fustini, Zoran Erlic, Michael Hugelshofer, Stefanos Voglis, Luca Regli, Diego Mazzatenta, Carlo Serra

**Affiliations:** a Machine Intelligence in Clinical Neuroscience (MICN) Laboratory, Department of Neurosurgery, Clinical Neuroscience Center, University Hospital Zurich, University of Zurich, Zurich, Switzerland; b IRCCS Istituto Delle Scienze Neurologiche di Bologna. Programma Neurochirurgia Ipofisi - Pituitary Unit, Bologna, Italy; c Department of Biomedical and Neuromotor Sciences (DIBINEM), University of Bologna, Italy; d Azienda USL di Bologna, Anatomic Pathology Unit, Bologna, Italy; e Department of Experimental, Diagnostic and Specialty Medicine (DIMES), University of Bologna, Italy; f University of Bologna, School of Medicine and Surgery, Bologna, Italy; g Azienda USL di Bologna, Bellaria Hospital, ENT Unit, Bologna, Italy; h Department of Endocrinology, Diabetology and Clinical Nutrition, University Hospital Zurich (USZ) and University of Zurich (UZH), Zurich, Switzerland

**Keywords:** Pituitary surgery, Adenoma, Outcome prediction, Machine learning, Transsphenoidal surgery

## Abstract

**Introduction:**

Gross total resection (GTR), Biochemical Remission (BR) and restitution of a priorly disrupted hypothalamus pituitary axis (new improvement, IMP) are important factors in pituitary adenoma (PA) resection surgery. Prediction of these metrics using simple and preoperatively available data might help improve patient care and contribute to a more personalized medicine.

**Research question:**

This study aims to develop machine learning models predicting GTR, BR, and IMP in PA resection surgery, using preoperatively available data.

**Material and methods:**

With data from patients undergoing endoscopic transsphenoidal surgery for PAs machine learning models for prediction of GTR, BR and IMP were developed and externally validated. Development was carried out on a registry from Bologna, Italy while external validation was conducted using patient data from Zurich, Switzerland.

**Results:**

The model development cohort consisted of 1203 patients. GTR was achieved in 207 (17.2%, 945 (78.6%) missing), BR in 173 (14.4%, 992 (82.5%) missing) and IMP in 208 (17.3%, 167 (13.9%) missing) cases. In the external validation cohort 206 patients were included and GTR was achieved in 121 (58.7%, 32 (15.5%) missing), BR in 46 (22.3%, 145 (70.4%) missing) and IMP in 42 (20.4%, 7 (3.4%) missing) cases. The AUC at external validation amounted to 0.72 (95% CI: 0.63–0.80) for GTR, 0.69 (0.52–0.83) for BR, as well as 0.82 (0.76–0.89) for IMP.

**Discussion and conclusion:**

All models showed adequate generalizability, performing similarly in training and external validation, confirming the possible potentials of machine learning in helping to adapt surgical therapy to the individual patient.

## Highlights

• **Outcome prediction in pituitary surgery using simple, preoperatively available data**.
• **Rigorous external validation of our machine learning prediction models**.
• **Adequate generalizability using multicenter data**.

## 1 Introduction

Pituitary adenomas (PA) constitute for roughly 15% of intracranial tumors and can be resected by transsphenoidal surgery (TSS) in the majority of cases (Thapar et al., 2001). TSS has been adopted as the ‘gold standard’ approach due to its minimal invasiveness with concomitant low morbidity and mortality (Kanter et al., 2005).

Many variables play a role in determining surgical and endocrinological outcomes of pituitary surgery, which have been analyzed in several publications (Lobatto et al., 2018; Zhou et al., 2017; Braileanu et al., 2019; Hensen et al., 1999). This burdens the clinicians with a multitude of variables to consider in surgical decision-making. In times when “big data” is easily accessible, machine learning (ML) – at least in theory – promises the ability to integrate all of these factors to better guide clinicians based on the individual characteristics of the patient (Obermeyer and Emanuel, 2016; Stumpo et al., 2022).

Using ML to predict the likelihood of endocrinological endpoints, such as biochemical remission (BR) or the restitution of a priorly disrupted hypothalamus pituitary (HP) axis (new improvement, IMP) as well as gross total resection (GTR) from simple preoperative data would be beneficial for clinicians and patients by leading to improved clinical decision-making and therefore improve patient outcome.

To date, none of the clinical prediction models for pituitary surgery outcomes have been externally validated (Stumpo et al., 2022). External validation is a critical step in evaluating the applicability of any model before introduction into clinical practice as internal validation and resampling techniques only allow for a very limited conclusion of the ultimate performance on new patients (generalization) (Collins et al., 2014). Therefore, we aimed to create externally validated, clinically applicable prediction models for anticipation of the beforementioned outcomes after TSS for PAs.

## 2 Methods

### 2.1 Overview

Prediction model development was carried out using data of patients that were treated with endoscopic TSS by the Department of Neurosurgery, IRCCS Institute of Neurological Sciences of Bologna. The models were trained to predict GTR, BR, and IMP respectively. External validation of the trained models was subsequently performed with patient data provided by the Department of Neurosurgery, University Hospital Zurich. In this study we adhere to methodology described in a previous publication but apply it to additional new data. (Zanier et al., 2021). All examinations were conducted adhering to the principles of transparent reporting of a multivariable prediction model for individual prognosis or diagnosis (TRIPOD) (Collins et al., 2015).

### 2.2 Ethical considerations

Patient data were treated according to the ethical standards of the Declaration of Helsinki and its amendments as approved by our institutional committee (Cantonal Ethics Committee Zürich, KEK St-V-Nr, 2015-0242) and the interhospital Ethical Committee of Bologna City (protocol CE17143, February 2018).

### 2.3 Data sources

This study was conducted using data from two centers, one of which was used for model training while data from the other center was applied to externally validate all models. Patients who underwent endoscopic TSS surgery for PA during the timeframe of August 1998 to January 2020 in Bologna as well as from July 2013 to May 2020 in Zurich were included in this study. All pre- and postoperative assessments as well as intraoperative procedures were carried out as specified in earlier publications (Maldaner et al., 2018; Serra et al., 2016). All patients included had to have information available for at least one of the three outcome measures and patients that underwent transcranial or combined procedures were excluded.

### 2.4 Outcomes

Classification machine learning models were trained for the prediction of GTR, BR and IMP as endpoints. GTR was strictly defined as an extent of resection of 100 The evaluation of the extent of resection was based on MRI images captured three months after the surgery and was conducted by a board-certified neurosurgeon with significant knowledge in pituitary imaging in collaboration with an experienced neuroradiologist. Furthermore, BR was defined as reduction of hormonal levels back into reference ranges, while IMP was defined as the restoration of one or more previously disrupted hypothalamus-pituitary axes into normal reference range of the respective hormones as specified by international guidelines (Giustina et al., 2020). Note that when calculating BR and IMP, additional treatment modalities such as medical and radiation therapy were taken into account. This is because withholding these essential treatments would be unethical.

### 2.5 Input variables

Gender, age, adenoma phenotype, Hardy score (sellar, suprasellar and parasellar) (Hardy and Vezina, 1976), Knosp classification (Knosp et al., 1993), number of disrupted hypothalamus-pituitary axes, preoperative level of TSH, GNRH and ACTH, nasoseptal flap and fascial reconstruction were selected as input variables. (Hardy and Vezina, 1976; Knosp et al., 1993). Both the neurosurgeon as well as the neuroradiologist independently assessed Knosp and Hardy scores based on the last preoperative MRI.

### 2.6 Model development

As previously described the prediction models were derived using data from Bologna and then externally validated on patient data from Zurich. Both datasets were shuffled randomly before being checked for equal class distribution. In a next step recursive feature elimination was applied to all initially available variables in order to arrive at a sparse model (Staartjes et al., 2022). In terms of model architecture, we applied support vector machines (SVMs), Random Forests (RFs) and Bagged Classification and Regression Trees (CARTs). The models were then trained and selected based on the area under the receiver operating curve (AUC) through 10 iterations of 10-fold cross validation. In parallel to this we also trained a k-nearest neighbour (KNN) algorithm, which allowed for any current and future imputation of missing data (Batista and Monard, 2003). Since our models are capable of providing continuous probabilities, we binarized the results based on the closest-to-(0,1)-criterion in order for model performance evaluations (Perkins and Schisterman, 2006). To evaluate discrimination, several metrics were employed, including the area under the curve (AUC), accuracy, sensitivity, specificity, positive predictive value (PPV), and negative predictive value (NPV). Nonparametric 95% confidence intervals (CI) of these metrics were computed. We also evaluated model calibration using the calibration curve intercept and slope. Finally variable importance was computed for each of the models in a universal AUC-based approach before being scaled from 0 to 100 (Kuhn, 2008). We carried out all our examinations using R version 4.0.2 (R Core Team, 2017).

## 3 Results

### 3.1 Patient characteristics

Overall, 1203 patients from Bologna were used in training the models, among whom 576 (47.9%) were male. The mean age amounted to 50.62 ± 16.05. GTR was accomplished in 207 (17.2%, 945 (78.6%) missing) and BR in 173 (14.4%, 992 (82.5%) missing) patients. IMP occurred in 208 (17.3%, 167 (13.9%) missing) cases. The external validation cohort (Zurich) consisted of 206 patients with mean age of 55.67 ± 16.80, 115 (55.8%) of them being male. IMP was recorded in 42 (20.4%, 7 (3.4%) missing) patients while GTR and BR was achieved for 121 (58.7%, 32 (15.5%) missing) and 46 (22.3%, 145 (70.4%) missing) patients respectively in the external validation group. An overview of the patient characteristics is given in Table 1.

**Table 1:** Patient characteristics and incidence of outcomes.

Variable	Cohort	
	Development (n = 1203)	External Validation (n = 206)
Male gender, n (%)	576 (47.9%)	115 (55.8%)
*No. missing*	*1 (0.1%)*	*1 (0.5%)*
Age [yrs.] Mean ± SD	50.62 ± 16.05	55.67 ± 16.80
*No. missing*	*0 (0.0%)*	*0 (0.0%)*
Phenotype, n (%)		
NFPA	306 (25.4%)	141 (68.4%)
ACTH-secreting	167 (13.9%)	6 (2.9%)
GH-secreting	315 (26.2%)	42 (20.4%)
PRL-secreting	158 (13.1%)	13 (6.3%)
FSH-secreting	235 (19.5%)	0 (0.0%)
TSH-secreting	10 (0.8%)	1 (0.5%)
Plurihormonal	12 (1.0%)	3 (1.5%)
*No. missing*	*0 (0.0%)*	*6 (2.9%)*
Hardy sellar, n (%)	1064 (88.4%)	198 (96.1%)
Grade 1	195 (16.2%)	25 (12.1%)
Grade 2	687 (57.1%)	59 (28.6%)
Grade 3	154 (12.8%)	23 (11.2%)
Grade 4	28 (2.3%)	91 (44.2%)
*No. missing*	*3 (0.2%)*	*6 (2.9%)*
Hardy suprasellar, n (%)	867 (72.1%)	161 (78.2%)
Grade A	624 (51.9%)	50 (24.3%)
Grade B	205 (17.0%)	71 (34.5%)
Grade C	38 (3.2%)	40 (19.4%)
*No. missing*	*1 (0.1%)*	*7 (3.4%)*
Hardy parasellar, n (%)	189 (15.7%)	57 (27.7%)
Grade D	21 (1.7%)	4 (1.9%)
Grade E	168 (14.0%)	53 (25.7%)
*No. missing*	*2 (0.2%)*	*5 (2.4%)*
Knosp classification, n (%)	558 (46.4%)	171 (83.0%)
Grade 1	206 (17.1%)	49 (23.8%)
Grade 2	156 (13.0%)	61 (29.6%)
Grade 3	143 (11.9%)	46 (22.3%)
Grade 4	53 (4.4%)	15 (7.3%)
*No. missing*	*2 (0.2%)*	*3 (1.5%)*
Number of disrupted hypothalamus pituitary axes Mean ± SD	0.74 ± 1.09	0.81 ± 1.02
*No. missing*	*310 (25.8%)*	*0 (0.0%)*
Fascial reconstruction, n (%)	29 (2.4%)	30 (14.6%)
*No. missing*	*705 (58.5%)*	*5 (2.4%)*
Nasoseptal flap reconstruction, n (%)	5 (0.4%)	9 (4.4%)
*No. missing*	*705 (58.5%)*	*0 (0.0%)*
Preop. TSH deficiency, n (%)	163 (13.5%)	66 (32.0%)
*No. missing*	*311 (25.9%)*	*0 (0.0%)*
Preop. ACTH deficiency, n (%)	138 (11.5%)	49 (23.8%)
*No. missing*	*478 (39.7%)*	*0 (0.0%)*
Preop. GNRH deficiency, n (%)	346 (28.8%)	51 (24.8%)
*No. missing*	*0 (0.0%)*	*0 (0.0%)*

Outcome	Cohort	
	Development (n = 1203)	External Validation (n = 206)
Gross total resection, n (%)	207 (17.2%)	121 (58.7%)
*No. missing*	*945 (78.6%)*	32 (15.5*%*)
Biochemical remission, n (%)	173 (14.4%)	46 (22.3%)
*No. missing*	*992 (82.5%)*	*145 (70.4%)*
New improvement, n (%)	208 (17.3%)	42 (20.4%)
*No. missing*	*167 (13.9%)*	*7 (3.4%)*

SD, standard deviation; IQR, interquartile range; NFPA, non-functioning pituitary adenoma; ACTH, adrenocorticotropic hormone; GH, growth hormone; PRL, prolactin, FSH follicle stimulating hormone; TSH, thyroid stimulating hormone.

### 3.2 Model performance

**Fig. 1:**
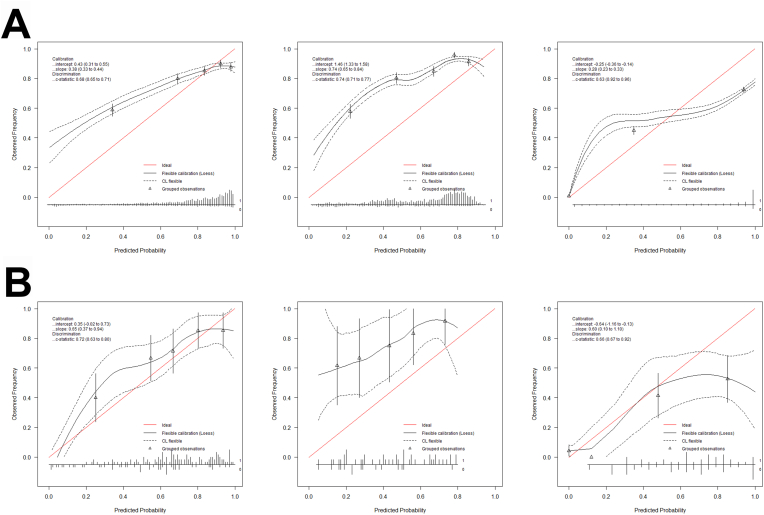
Calibration curves of the prediction models during training (A) and external validation (B). Within each row, gross total resection (GTR) is shown to the left, followed by biochemical remission (BR) in the middle and improvements (IMP) on the right side. The predicted probabilities for the outcomes are distributed into five equally sized groups and contrasted with the observed frequencies of the outcomes. Calibration intercept and slope are then calculated. A perfectly calibrated model has a calibration intercept of 0 and slope of 1. The calibration intercept is influenced by the frequency of the outcome of interest in a certain population. Metrics are provided with bootstrapped 95% confidence intervals.

An overview of model performances, including calibration metrics and training performance, is supplied in Table 2 and the related calibration plots are provided in Fig. 1.

**Table 2:** Quantitative evaluation of discrimination and calibration of the prediction models.

Outcome	Gross total resection		Biochemical Remission		New Improvement	
Type of model	Random Forest		Support Vector Machine		Bagged CART	
Metric	Development (n = 285)	External Validation (n = 174)	Development (n = 211)	External Validation (n = 61)	Development (n = 1036)	External Validation (n = 199)
Discrimination						
AUC	0.68 (0.65–0.71)	0.72 (0.63–0.80)	0.74 (0.71–0.77)	0.69 (0.52–0.83)	0.94 (0.93–0.94)	0.82 (0.76–0.89)
Accuracy	0.68 (0.66–0.70)	0.54 (0.47–0.61)	0.65 (0.63–0.67)	0.46 (0.33–0.59)	0.88 (0.88–0.89)	0.75 (0.69–0.81)
Sensitivity	0.70 (0.68–0.72)	0.41 (0.32–0.51)	0.63 (0.61–0.65)	0.30 (0.18–0.43)	0.95 (0.94–0.96)	0.88 (0.78–0.97)
Specificity	0.59 (0.55–0.63)	0.83 (0.72–0.92)	0.73 (0.68–0.77)	0.93 (0.78–1.00)	0.87 (0.86–0.88)	0.72 (0.65–0.79)
PPV	0.87 (0.86–0.89)	0.85 (0.75–0.93)	0.91 (0.90–0.93)	0.93 (0.79–1.00)	0.64 (0.63–0.66)	0.46 (0.35–0.57)
NPV	0.33 (0.30–0.36)	0.38 (0.29–0.47)	0.30 (0.27–0.33)	0.30 (0.17–0.45)	0.99 (0.98–0.99)	0.96 (0.92–0.99)
F1 Score	0.42	0.52	0.43	0.46	0.92	0.82
Calibration						
Intercept	0.43 (0.31–0.55)	0.35 (−0.02 – 0.73)	1.46 (1.33 – 1-58)	1.75 (1.11–2.38)	−0.25 (−0.36 to −0.14)	−0.64 (−1.16 to −0.13)
Slope	0.38 (0.33–0.44)	0.65 (0.37–0.94)	0.74 (0.71–0.77)	0.65 (0.04–1.27)	0.28 (0.23–0.33)	0.60 (0.10–1.10)
Threshold	0.75		0.61		0.10	

Metrics are presented along with their 95% confidence intervals derived using bootstrapping.

AUC, area under the curve; PPV, positive predictive value; NPV, negative predictive value; CART, classification and regression trees.

### 3.3 Gross total resection (GTR)

In external validation, the Random Forest model used to predict GTR yielded an AUC of 0.72 (0.63–0.80). The sensitivity and the specificity amounted to 0.41 (0.32–0.51) and 0.83 (0.72–0.92), respectively. A positive predictive value (PPV) of 0.85 (0.75–0.93) was reached.

### 3.4 Biochemical remission (BR)

On the external validation cohort, the BR prediction model (Support Vector Machine) achieved an AUC of 0.69 (0.52–0.83). Sensitivity and a specificity amounted to 0.30 (0.18–0.43) and 0.93 (0.78–1.00) respectively. A PPV of 0.93 (0.79–1.00) was obtained.

### 3.5 Improvement of one or more HP axes (IMP)

During external validation the bagged CART model trained to predict IMP attained an AUC of 0.82 (0.76–0.89), whereas sensitivity reached 0.88 (0.78–0.97) and a specificity of 0.72 (0.65–0.79) was registered. A NPV of 0.96 (0.92–0.99) was achieved.

### 3.6 Variable importance

**Fig. 2:**
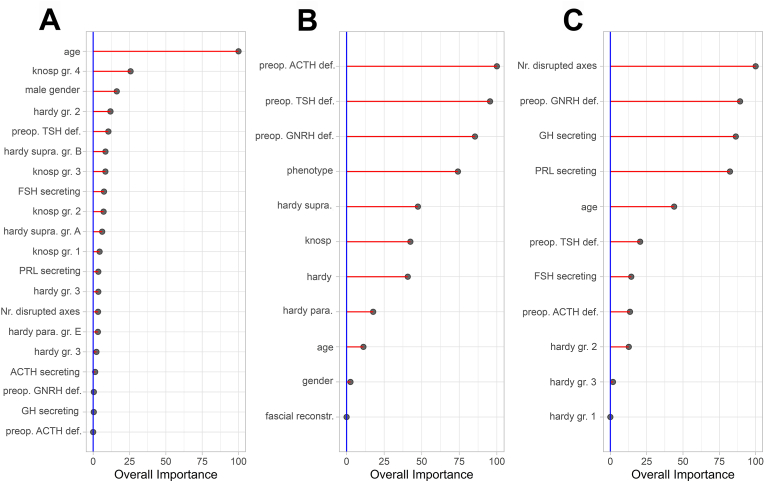
Variable importance based on AUC for the three models, with importance values scaled from 0 to 100. Gross Total Resection (A), Biochemical Remission (B), and New Improvements (C).

Fig. 2 and Table 3 provide a synopsis of the variable importances for each of the developed models. Knosp classification and patient age contributed most to the prediction of GTR, while a preoperative deficit of ACTH and TSH contributed most to the prediction of BR. Lastly, the number of disrupted hypothalamus-pituitary axes and GNRH deficit had the greatest influence on prediction of improvement of one or more HP axes (IMP).

**Table 3:** AUC-based relative variable importance of the prediction models.

Variable	Gross total resection	Biochemical remission	New improvement
Male gender	16.14	2.56	*
Age	100.00	11.19	43.90
Phenotype		74.11	
NFPA-secreting	0.00		0.00
ACTH-secreting	1.38		0.00
GH-secreting	0.39		86.34
PRL-secreting	3.51		82.32
FSH-secreting	7.42		14.37
TSH-secreting	0.00		0.00
Plurihormonal	0.00		0.00
Hardy sellar		40.71	
Grade 1	3.50		0.00
Grade 2	11.85		12.65
Grade 3	2.22		1.78
Grade 4	0.00		0.00
Hardy suprasellar		47.47	*
Grade A	6.21		
Grade B	8.42		
Grade C	0.00		
Hardy parasellar		17.70	*
Grade D	0.00		
Grade E	3.23		
Knosp classification		42.46	*
Grade 1	4.40		
Grade 2	7.17		
Grade 3	8.38		
Grade 4	25.66		
Number of disrupted hypothalamus pituitary axes	3.33	*	100.00
Fascial reconstruction	*	0.00	*
Nasoseptal flap reconstruction	0.00	*	*
Preop. TSH deficiency	10.41	95.50	20.50
Preop ACTH deficiency	0.00	100.00	13.50
Preop. GNRH deficiency	0.44	85.43	89.28

Asterisks (*) denote variable importances of 0.00, indicating that the variable was not included in the final model.

NFPA, non-functioning pituitary adenoma; ACTH, adrenocorticotropic hormone; GH, growth hormone; PRL, prolactin, FSH follicle stimulating hormone; TSH, thyroid stimulating hormone.

### 3.7 Deployment

We integrated our prediction models into a complimentary web application accessible at https://neurosurgery.shinyapps.io/pituicalc.

## 4 Discussion

With multicenter data of over 250 to 1200 patients, depending on the model, we developed and rigorously externally validated clinical prediction models that demonstrated moderate ability to predict GTR, BR and IMP following TSS for PA. Calibration performance was adequate. Although generalizable models were derived, their added value in clinical practice needs to be critically evaluated and their performance compared to that of human experts.

Surgical and especially endocrinological outcomes after pituitary surgery are notoriously hard to predict preoperatively (Sorba et al., 2021; Fatemi et al., 2008; Dhandapani et al., 2016). Currently, physicians approach questions like “*How likely is it that you can remove the tumor completely?*” by citing numbers derived from case series in the literature or from their own case series. However, this can hardly be considered “precision medicine”. Existing approaches to make prediction of surgical and endocrinological outcomes more individualized include the use of classifications, e.g., the Knosp classification or the Zurich Pituitary score for GTR, to stratify patients into large groups with different risk-benefit profiles (Knosp et al., 1993; Serra et al., 2018). Still, this does not allow any statement of each particular patient's risk-benefit profile and can hardly be considered precise on an individual level. It is exactly here that clinical prediction models – including ML techniques – have promised improvements by integrating relatively complex sets of variables, enabling personalized predictions for each individual patient (Obermeyer and Emanuel, 2016; Stumpo et al., 2022). In some cases, models have even been shown to outperform human medical experts (Senders et al., 2018). Nevertheless, some factors obviously cannot be accounted for by any model or would simply be too cumbersome to collect, thus preventing efficient clinical use. Clinical prediction models should therefore be considered merely as assistive tools that may aid in physicians' decision-making process, but should never replace the literature, imaging, and medical expertise.

Using data from a single center and externally validating our models on data from a separate center, we have generated clinical prediction models for GTR, BR and IMP. A specific goal of our study was to keep the amount and the complexity of preoperative variables required as inputs to a clinically applicable minimum. On one hand inclusion of actual medical imaging files or sophisticated measurement methods might increase predictive performance but on the other hand this would likely preclude any wide-spread clinical application.

Predicting endocrinological outcomes or GTR is only an initial step that shows the potentials machine learning offers and its potential impact on clinical decision making. Important complicating factors like pituitary apoplexy could be included into the prediction models or risks of postoperative complications like transient hyponatremia, a primary cause for recurrent hospitalization after TSS PA resection, could be predicted (Zoli et al., 2016, 2017). To date these still are immensely difficult to foresee, but the power of machine learning to deduct simple information out of complex data might help with the management of such complications.

The performance measures obtained at external validation show that predicting postoperative results based on simple preoperative data is quite challenging. Nonetheless, our models predicted the outcomes mentioned above with acceptable calibration and adequate discrimination.

There have been previous attempts to derive prediction models for outcomes after pituitary surgery. However, to the best of the authors’ knowledge, almost none of the models for outcomes after surgery for PA have been externally validated. Only two externally validated prediction models exist, but these are targeted only towards surgery for GH-secreting PA. Qiao et al. (2021) predicted early BR after surgery in patients with acromegaly, with externally validated AUCs of 0.77–0.85. Likewise, we previously (Zanier et al., 2021) predicted BR, cerebrospinal fluid leaks, and GTR with AUCs of 0.63–0.77 at external validation. Compared to these studies our model performances lie within a similar range. While these two models are the only ones externally validated, indeed, multiple models without external validation have been published for pituitary surgery in general. Stumpo et al. (2022) recently reviewed the literature on machine learning in pituitary surgery. A model developed by Hollon et al. (2018) demonstrated AUCs ranging between 0.80 and 0.85 based on internal validation. Fan et al. (2019) used radiomic data to predict BR among hormone-secreting adenomas demonstrating an internal validation AUC of 0.81. Staartjes et al., 2018, 2019 demonstrated that neural networks classified GTR likelihood more accurately than the Knosp classification or a logistic regression model with an internally validated AUC of 0.87 and that even intraoperative cerebrospinal fluid leaks can be predicted with comparable performance.

Without external validation, achieving relatively good performance in cross-validation or in a held-out internal validation cohort is fairly straightforward, but this does not in any way imply a similar model performance when applied on other cohorts (Staartjes and Kernbach, 2020).

The performance of human experts in predicting these outcomes has not yet been evaluated systematically, but it is likely to be inferior to the performance measures observed in this study. In other domains of neurosurgery, it has been shown that e.g., new neurological deficits or outcomes after spine surgery are only poorly predicted by neurosurgeons (Sagberg et al., 2017). Compared to current approaches, our models are at least able to provide an objective benchmark of expected outcomes on an individual level. Such objective benchmarks can be useful when comparing quality between centers, when evaluating scientific research, or simply as a “second opinion”. In patient cases where the indication to undergo surgery is not as clear cut, a model predicting a rather high chance of GTR and BR might be of help in strengthening the decision to lead through with surgical intervention. In contrast to that, prediction of a low chance of GTR, also considering other established sources of information, might lead to changes surgical indications. Taking these points into account, we do not recommend using clinical prediction models as decisive components of clinical decision making on their own.

Our models have been integrated into a free available web application. We encourage physicians to attempt implementation into clinical practice taking however into account the developmental stage and limitations of the models.

## 5 Limitations

There are limitations arising from any prediction model, including the ones we built in this study: Countless immeasurable and measurable factors such as surgeon experience, caseload, postoperative management protocols, and others limit the generalizability of any parsimonious prediction model (Sorba et al., 2021; Barker et al., 2003). This means that our models are perhaps ill-suited for institutions that use wholly different treatment and postoperative management methods and may give erroneous results. Furthermore, if the input data fall outside the range of data which the model was trained on, it will unlikely provide accurate information (extrapolation).

Despite having externally validated our models, which shows that they are already generalizable, a multicenter training dataset could help to drastically improve generalizability of our predictive models due to factors such as the ones mentioned previously. The availability of more data preoperatively, such as sodium and potassium concentrations, peripheral hormone levels or surrogates like the fT4/TSH quotient, could also improve performance, but collecting and entering more data into the web-application may be cumbersome.

While the Knosp and Hardy scores are still commonly determined in clinical practice, they have a rather low interrater reliability for intermediate scores (Mooney et al., 2017a, 2017b). Inclusion of simpler and more dependable scoring systems could potentially lead to additional enhancements in model performance.

Finally, although our cohort included a very decent amount of over 1200 PA patients - one of the largest cohorts in current literature – due to its retrospective nature, this study encompasses a significant number of patients with missing information for some of the outcomes. In that respect, a higher number of training samples would likely further improve model performance and should be considered in the future development.

## 6 Conclusion

Based on a large cohort of patients with PAs, prediction of GTR, BR and IMP was feasible with moderate to good performance at external validation, thereby confirming generalizability. Although outcomes after pituitary surgery, especially endocrinological outcomes, are hard to predict, based on our results the role of clinical prediction models as assistive tools in surgical decision making can be reinforced.

## Funding

This research did not receive funding in any form.

## Abstract publication

A previous version of this study's abstract has been published as part of EANS congress 2022 under doi.org/10.1016/j.bas.2022.101539.

## Declaration of interests

The authors declare that they have no known competing financial interests or personal relationships that could have appeared to influence the work reported in this paper.

## References

[bib1] Barker F.G., Klibanski A., Swearingen B. (2003). Transsphenoidal surgery for pituitary tumors in the United States, 1996-2000: mortality, morbidity, and the effects of hospital and surgeon volume. J. Clin. Endocrinol. Metab..

[bib2] Batista G.E.A.P.A., Monard M.C. (2003). An analysis of four missing data treatment methods for supervised learning. Appl. Artif. Intell..

[bib3] Braileanu M., Hu R., Hoch M.J., Mullins M.E., Ioachimescu A.G., Oyesiku N.M., 2nd Pappy A., Saindane A.M. (2019). Pre-operative MRI predictors of hormonal remission status post pituitary adenoma resection. Clin. Imag..

[bib4] Collins G.S., de Groot J.A., Dutton S., Omar O., Shanyinde M., Tajar A., Voysey M., Wharton R., Yu L.-M., Moons K.G., Altman D.G. (2014). External validation of multivariable prediction models: a systematic review of methodological conduct and reporting. BMC Med. Res. Methodol..

[bib5] Collins G.S., Reitsma J.B., Altman D.G., Moons K.G.M. (2015). Transparent reporting of a multivariable prediction model for individual prognosis or diagnosis (TRIPOD): the TRIPOD statement. Ann. Intern. Med..

[bib6] Dhandapani S., Singh H., Negm H.M., Cohen S., Anand V.K., Schwartz T.H. (2016). Cavernous sinus invasion in pituitary adenomas: systematic review and pooled data meta-analysis of radiologic criteria and comparison of endoscopic and microscopic surgery. World Neurosurg..

[bib7] Fan Y., Liu Z., Hou B., Li L., Liu X., Liu Z., Wang R., Lin Y., Feng F., Tian J., Feng M. (2019). Development and validation of an MRI-based radiomic signature for the preoperative prediction of treatment response in patients with invasive functional pituitary adenoma. Eur. J. Radiol..

[bib8] Fatemi N., Dusick J.R., Mattozo C., McArthur D.L., Cohan P., Boscardin J., Wang C., Swerdloff R.S., Kelly D.F. (2008). Pituitary hormonal loss and recovery after transsphenoidal adenoma removal. Neurosurgery.

[bib9] Giustina A., Barkhoudarian G., Beckers A., Ben-Shlomo A., Biermasz N., Biller B., Boguszewski C., Bolanowski M., Bollerslev J., Bonert V., Bronstein M.D., Buchfelder M., Casanueva F., Chanson P., Clemmons D., Fleseriu M., Formenti A.M., Freda P., Gadelha M., Geer E., Gurnell M., Heaney A.P., Ho K.K.Y., Ioachimescu A.G., Lamberts S., Laws E., Losa M., Maffei P., Mamelak A., Mercado M., Molitch M., Mortini P., Pereira A.M., Petersenn S., Post K., Puig-Domingo M., Salvatori R., Samson S.L., Shimon I., Strasburger C., Swearingen B., Trainer P., Vance M.L., Wass J., Wierman M.E., Yuen K.C.J., Zatelli M.C., Melmed S. (2020). Multidisciplinary management of acromegaly: a consensus. Rev. Endocr. Metab. Disord..

[bib10] Hardy J., Vezina J.L. (1976). Transsphenoidal neurosurgery of intracranial neoplasm. Adv. Neurol..

[bib11] Hensen J., Henig A., Fahlbusch R., Meyer M., Boehnert M., Buchfelder M. (1999). Prevalence, predictors and patterns of postoperative polyuria and hyponatraemia in the immediate course after transsphenoidal surgery for pituitary adenomas. Clin. Endocrinol..

[bib12] Hollon T.C., Parikh A., Pandian B., Tarpeh J., Orringer D.A., Barkan A.L., McKean E.L., Sullivan S.E. (2018). A machine learning approach to predict early outcomes after pituitary adenoma surgery. Neurosurg. Focus.

[bib13] Kanter A.S., Dumont A.S., Asthagiri A.R., Oskouian R.J., Jane J.A., Laws E.R. (2005). The transsphenoidal approach. A historical perspective. Neurosurg. Focus.

[bib14] Knosp E., Steiner E., Kitz K., Matula C. (1993). Pituitary adenomas with invasion of the cavernous sinus SpaceA magnetic resonance imaging classification compared with surgical findings. Neurosurgery.

[bib15] Kuhn M. (2008). Building predictive models in *R* using the caret package. J. Stat. Software.

[bib16] Lobatto D.J., de Vries F., Najafabadi A.H.Z., Pereira A.M., Peul W.C., Vlieland T.P.M.V., Biermasz N.R., van Furth W.R. (2018). Preoperative risk factors for postoperative complications in endoscopic pituitary surgery: a systematic review. Pituitary.

[bib17] Maldaner N., Serra C., Tschopp O., Schmid C., Bozinov O., Regli L. (2018). Modernes Management von Hypophysenadenomen – gegenwärtiger Stand in Diagnostik, Therapie und Nachsorge. Praxis.

[bib18] Mooney M.A., Hardesty D.A., Sheehy J.P., Bird C.R., Chapple K., White W.L., Little A.S. (2017). Rater reliability of the Hardy classification for pituitary adenomas in the magnetic resonance imaging era. J. Neurol. Surg. Part B Skull Base.

[bib19] Mooney M.A., Hardesty D.A., Sheehy J.P., Bird R., Chapple K., White W.L., Little A.S. (2017). Interrater and intrarater reliability of the Knosp scale for pituitary adenoma grading. J. Neurosurg..

[bib20] Obermeyer Z., Emanuel E.J. (2016). Predicting the future - big data, machine learning, and clinical medicine. N. Engl. J. Med..

[bib21] Perkins N.J., Schisterman E.F. (2006). The inconsistency of “optimal” cutpoints obtained using two criteria based on the receiver operating characteristic curve. Am. J. Epidemiol..

[bib22] Qiao N., Shen M., He W., He M., Zhang Z., Ye H., Li Y., Shou X., Li S., Jiang C., Wang Y., Zhao Y. (2021). Machine learning in predicting early remission in patients after surgical treatment of acromegaly: a multicenter study. Pituitary.

[bib23] (2017). R Core Team. https://www.R-project.org/.

[bib24] Sagberg L.M., Drewes C., Jakola A.S., Solheim O. (2017). Accuracy of operating neurosurgeons' prediction of functional levels after intracranial tumor surgery. J. Neurosurg..

[bib25] Senders J.T., Arnaout O., Karhade A.V., Dasenbrock H.H., Gormley W.B., Broekman M.L., Smith T.R. (2018). Natural and artificial intelligence in neurosurgery: a systematic review. Neurosurgery.

[bib26] Serra C., Burkhardt J.-K., Esposito G., Bozinov O., Pangalu A., Valavanis A., Holzmann D., Schmid C., Regli L. (2016). Pituitary surgery and volumetric assessment of extent of resection: a paradigm shift in the use of intraoperative magnetic resonance imaging. Neurosurg. Focus.

[bib27] Serra C., Staartjes V.E., Maldaner N., Muscas G., Akeret K., Holzmann D., Soyka M.B., Schmid C., Regli L. (2018). Predicting extent of resection in transsphenoidal surgery for pituitary adenoma. Acta Neurochir..

[bib28] Sorba E.L., Staartjes V.E., Voglis S., Tosic L., Brandi G., Tschopp O., Serra C., Regli L. (2021). Diabetes insipidus and syndrome of inappropriate antidiuresis (SIADH) after pituitary surgery: incidence and risk factors. Neurosurg. Rev..

[bib29] Staartjes V.E., Kernbach J.M. (2020). Significance of external validation in clinical machine learning: let loose too early?. Spine J. Off. J. North Am. Spine Soc..

[bib30] Staartjes V.E., Serra C., Muscas G., Maldaner N., Akeret K., van Niftrik C.H.B., Fierstra J., Holzmann D., Regli L. (2018). Utility of deep neural networks in predicting gross-total resection after transsphenoidal surgery for pituitary adenoma: a pilot study. Neurosurg. Focus.

[bib31] Staartjes V.E., Zattra C.M., Akeret K., Maldaner N., Muscas G., Bas van Niftrik C.H., Fierstra J., Regli L., Serra C. (2019). Neural network-based identification of patients at high risk for intraoperative cerebrospinal fluid leaks in endoscopic pituitary surgery. J. Neurosurg..

[bib32] Staartjes V., Kernbach J.M., Stumpo V., van Niftrik C.H.B., Serra C., Regli L. (2022). Mach. Learn. Clin. Neurosci. Found. Clin. Appl..

[bib33] Stumpo V., Staartjes V., Regli L., Serra C. (2022). Mach. Learn. Clin. Neurosci. Found. Clin. Appl..

[bib34] Thapar K., Kovacs K., Scheithauer B., Lloyd R.V. (2001). Diagnosis and Management of Pituitary Tumors.

[bib35] Zanier O., Zoli M., Staartjes V.E., Guaraldi F., Asioli S., Rustici A., Marino Picciola V., Pasquini E., Faustini-Fustini M., Erlic Z., Regli L., Mazzatenta D., Serra C. (2021). Machine learning-based clinical outcome prediction in surgery for acromegaly. Endocrine.

[bib36] Zhou Q., Yang Z., Wang X., Wang Z., Zhao C., Zhang S., Li P., Li S., Liu P. (2017). Risk factors and management of intraoperative cerebrospinal fluid leaks in endoscopic treatment of pituitary adenoma: analysis of 492 patients. World Neurosurg..

[bib37] Zoli M., Mazzatenta D., Faustini-Fustini M. (2016). Transient delayed hyponatremia after transsphenoidal surgery: attempting to enlighten the epidemiology and management of a still-obscure complication. World Neurosurg..

[bib38] Zoli M., Milanese L., Faustini-Fustini M., Guaraldi F., Asioli S., Zenesini C., Righi A., Frank G., Foschini M.P., Sturiale C., Pasquini E., Mazzatenta D. (2017). Endoscopic endonasal surgery for pituitary apoplexy: evidence on a 75-case series from a tertiary care center. World Neurosurg..

